# Second Attempt for Patent Ductus Arteriosus (PDA) Closure: Room for Acetaminophen? A Retrospective Single-Center Experience at Gaslini Children’s Hospital

**DOI:** 10.3390/children12050577

**Published:** 2025-04-29

**Authors:** Samuele Caruggi, Andrea Calandrino, Gaia Cipresso, Marcella Battaglini, Paolo Massirio, Francesco Vinci, Irene Bonato, Chiara Andreato, Federica Mela, Lorenzo Curcio, Alessandro Parodi, Luca Antonio Ramenghi

**Affiliations:** 1 Neonatal Intensive Care Unit, Department of Maternal and Neonatal Health, IRCCS Giannina Gaslini Institute, 16147 Genoa, Italy; samuelecaruggi@gaslini.org (S.C.); gaiacipresso@gaslini.org (G.C.); marcellabattaglini@gaslini.org (M.B.); paolomassirio@gaslini.org (P.M.); francescovinci@gaslini.org (F.V.); irenebonato@gaslini.org (I.B.); chiaraandreato@gaslini.org (C.A.); alessandroparodi@gaslini.org (A.P.); lucaramenghi@gaslini.org (L.A.R.); 2 Department of Neuroscience, Rehabilitation, Ophthalmology, Genetics, Maternal and Child Health, University of Genoa, 16132 Genoa, Italy; 6552206@studenti.unige.it (F.M.); lorenzo.curcio@edu.unige.it (L.C.)

**Keywords:** hsPDA, acetaminophen, ibuprofen

## Abstract

**Background**: The diagnosis of hemodynamically significant patent ductus arteriosus (hsPDA) occurs in 55% of very low birth weight (VLBW) preterm infants. There is no agreement on the best approach to ensure a quick hsPDA closure. Drug treatment of hsPDA fails in approximately 20% of cases with an increasing risk of prolonged ventilation, BPD, and NEC, as well as the need for surgical duct ligation. This study aims to highlight the efficacy of ibuprofen versus acetaminophen in the case of a second cycle of medical therapy after the failure of the first pharmacological approach for hsPDA closure. **Methods**: Every VLBW infant admitted to our NICU and treated for hsPDA was included in our retrospective research. Information about the clinical course, hsPDA diagnosis and treatment, and common complications associated with preterm birth was collected. A comparison was made between patients treated with acetaminophen or ibuprofen to assess effectiveness in hsPDA closing. **Results**: A total of 286 VLBW infants were included. First-course ibuprofen was effective in 87 of 115 infants (75.7%) treated, acetaminophen in 138 of 171 (80.7%). Second-course therapy with ibuprofen was effective in 62.5% of the patients, while acetaminophen was effective in 69.2%. No statistically significant difference was observed in the first-course and second-course success rates. **Conclusions**: This study confirms that acetaminophen is not inferior to ibuprofen in the closure of hsPDA in VLBW infants. Our data demonstrate that a second course of medical therapy after the failure of the first course could help close the majority of hsPDA cases without surgery.

## 1. Introduction

Managing patent ductus arteriosus (PDA) in extremely premature infants is critical to neonatal care [1].

A hemodynamically significant patent ductus arteriosus (hsPDA) is associated with an increased risk of prolonged ventilation, bronchopulmonary dysplasia (BPD) [2,3,4], necrotizing enterocolitis (NEC) [5], severe brain lesions such as periventricular leukomalacia (PVL) and intraventricular hemorrhage (IVH) [6], increased mortality, and varying degrees of neurodevelopmental impairment (NDI) [3,7,8].

Approximately 55% of very low birth weight infants (VLBW infants born at <1500 g), 80% of infants born before 28 weeks gestation, and 90% of those born at 24 weeks [1,9,10] develop hsPDA, necessitating closure to alleviate a clinically significant left-to-right shunt causing systemic hypoperfusion and pulmonary overcirculation [7].

Significant advancements have occurred in the pharmacological treatment of hsPDA, but the optimal timing and strategies for its management are still debated.

Currently, ibuprofen and acetaminophen are the first-line pharmacotherapy, gradually replacing indomethacin, which was previously considered the standard treatment [10].

A recent Cochrane review demonstrated that ibuprofen has similar efficacy to indometacin in achieving hsPDA closure [11]. Due to the reduced risk of NEC and transient renal failure associated with ibuprofen compared to indomethacin, it is often preferred as the first-line drug. To achieve optimal blood levels, regardless of gestational age, three doses of ibuprofen, administered at 24 h intervals (10 mg/kg on the first day and 5 mg/kg on days 2 and 3), are recommended for newborns younger than 70 h, as described by Hirt in 2008 [12].

More recently, acetaminophen has gained popularity as a therapy for hsPDA closure, particularly in cases where COX inhibitors are contraindicated or ineffective [10,12,13,14,15]. Several studies suggest that acetaminophen is as effective as ibuprofen, with a lower risk of gastrointestinal bleeding and lower serum creatinine levels [8,13,14,15,16,17]. A recent study found no significant differences in the composite outcome of death or BPD among highly premature infants treated with either acetaminophen or COX inhibitors for hsPDA closure [18]. The standard dosage of acetaminophen is 15 mg/kg every 6 h. The treatment duration reported in the literature varies between 3 and 7 days [10,17].

Although ibuprofen and acetaminophen have demonstrated similar success rates in PDA closure (70–80%) [9,13], a significant proportion of PDAs fail to close after the initial course of medical therapy, necessitating a second drug therapy and, potentially, surgical intervention [8,9,19,20].

This retrospective study aims to enrich the existing literature regarding the pharmacological treatment of hsPDA.

While previous studies have compared the efficacy of ibuprofen and acetaminophen as first-line treatments for hsPDA, this retrospective study uniquely focuses on the comparative effectiveness of these agents as a second-line therapy following initial treatment failure. We further aimed to investigate whether specific patient characteristics influence the outcome of second-line treatment with either drug.

## 2. Materials and Methods

This is a retrospective, single-center study conducted on very low birth weight (VLBW) preterm infants admitted to the Neonatal Intensive Care Unit (NICU) of IRCCS Gaslini Children’s Hospital over ten years. A retrospective cohort was selected from all VLBW infants admitted to our NICU between 1 January 2015 and 31 December 2024. The inclusion criteria were admission within 6 h of birth and a diagnosis of hemodynamically significant patent ductus arteriosus (hsPDA) requiring treatment.

The exclusion criteria included significant congenital malformations, fetal hydrops, metabolic diseases, congenital heart disease, chromosomal disorders, and antenatal infections (e.g., cytomegalovirus infection). VLBW infants admitted to our NICU more than 6 h after birth were excluded. Patients who died before or during treatment were also excluded. In our NICU, ibuprofen was the first-line therapeutic choice for hsPDA closure until 2019.

Subsequently, acetaminophen replaced ibuprofen due to lower associated risks [8], becoming the preferred treatment for PDA closure from 2019 onwards. Consequently, we compared patients treated with these two different drugs. Our internal protocol for managing PDA includes a pediatric cardiologist’s initial functional and anatomical assessment to establish structural normality, measure the PDA diameter, and assess the direction of ductal shunting within 24 h of life. The protocol mandates a follow-up evaluation by a pediatric cardiologist 72 h after initiating PDA therapy to evaluate treatment efficacy. Further functional cardiac ultrasound evaluations were performed as needed.

Treatment for PDA was initiated upon the diagnosis of hsPDA. The criteria for defining hsPDA included PDA diameter, flow pattern, and the extent of volume overload. Therapy was typically initiated when the PDA diameter was ≥1.6 mm in the presence of a left atrial-to-aortic root ratio ≥ 1.6 or a non-restrictive left-to-right ductal shunt pattern (diastolic velocity < 50% of peak systolic velocity) [10,19,21,22].

Acetaminophen was administered 15 mg/kg intravenously four times daily for five days. Ibuprofen was administered intravenously for three days, with 10 mg/kg on the first and 5 mg/kg on the second and third days. Successful treatment was defined as ductal closure, characterized by the absence of flow across the ductus arteriosus.

Treatment failure was defined as the persistence of hsPDA after the therapeutic regimen’s completion or the ductus arteriosus reopening within 48 h of treatment cessation. The echocardiographic parameters were consistent with those previously described. Deviation from the standard treatment duration was also considered to represent treatment failure. In cases of treatment failure, a second course of either ibuprofen or acetaminophen was typically attempted, following consultation with a pediatric cardiologist. In the event of persistent failure following a second course of pharmacological therapy, the multidisciplinary team convened to determine whether to proceed with surgical ductal closure or to continue medical management. For each enrolled infant, data were collected on birth weight, gestational age at birth, antenatal factors such as intrauterine growth restriction (IUGR), and postnatal clinical outcomes, including necrotizing enterocolitis (NEC), retinopathy of prematurity (ROP), and bronchopulmonary dysplasia (BPD). All VLBW infants admitted to our NICU underwent brain magnetic resonance imaging (MRI) at term-equivalent age to assess brain development and prematurity-related lesions.

Descriptive statistical analyses were performed for the two cohorts. Continuous variables were presented as means ± standard deviations (SDs) or medians with interquartile ranges (IQRs), as appropriate. Categorical variables were presented as absolute frequencies and percentages.

The normality of continuous variable distributions was assessed graphically using the Shapiro–Wilk test where indicated. Comparisons of categorical variables between subgroups were performed using Fisher’s exact test. In contrast, continuous variables were compared using either the Mann–Whitney U test or Student’s *t*-test, depending on the distribution. A *p*-value of <0.05 was considered statistically significant, and all *p*-values were two-tailed. Statistical analysis was conducted using Jamovi^®^ software v. 2.6.17, an interface built upon the R statistical programming language v. 4.4.2.

## 3. Results

During the study period, 711 VLBW preterm infants were admitted to our Neonatal Intensive Care Unit. Of these, 286 patients met the inclusion criteria and were subsequently enrolled in the analysis. The selected patients were stratified into two cohorts, delineated by the initial therapeutic regimen administered for the closure of hsPDA.

Table 1 presents the characteristics of patients treated for hsPDA, stratified by the type of medication used. Despite the observation of a lower gestational age (GA) and birth weight (BW) in one of the two cohorts, no statistically significant differences were observed in the incidence of complications between the two groups, except for NEC incidence, which was lower in the acetaminophen group.

Table 2 presents the data of patients requiring a second course of pharmacological therapy following the failure of the first. No significant differences were detected. Table 3 compares the characteristics of patients who achieved hsPDA closure after the first drug course with those who experienced treatment failure. The median gestational age (26 + 5 weeks vs. 28 + 2 weeks) and median birth weight (880 g vs. 1035 g) were significantly lower in patients who failed the initial pharmacological therapy. Patients who experienced hsPDA closure failure following the initial course of medical treatment demonstrated a substantially higher incidence of BPD (65% vs. 36%, *p* < 0.05) and treated ROP (37% vs. 21%, *p* < 0.05) compared to those who achieved successful hsPDA closure after the first course of treatment.

The initial ibuprofen regimen (total duration: 3 days; 10 mg/kg on day 1, 5 mg/kg on days 2 and 3) achieved ductal closure in 87 of 115 infants (75.7%). The initial acetaminophen regimen (15 mg/kg intravenously four times daily for 5 days) achieved ductal closure in 138 out of 171 infants (80.7%).

Second-line therapy with ibuprofen resulted in ductal closure in 20 out of 32 infants (62.5%), while second-line acetaminophen achieved closure in 20 of 29 infants (69.2%). No statistically significant difference was observed in the success rates of either the first-line or second-line therapies (Table 2).

Considering only patients who received second-line pharmacological therapy after first-course failure, no statistically significant differences were observed in gestational age, birth weight, BPD, necrotizing enterocolitis (NEC), treated ROP, spontaneous intestinal perforation, or brain lesions between patients who also failed the second attempt at closure and those who achieved successful closure after the second course. Of the 21 patients who failed second-line therapy, 16 underwent surgical closure, 2 achieved successful closure with fluid restriction and indomethacin, 1 with ibuprofen, and 2 with acetaminophen.

Focusing solely on second-line drug therapy, 61 patients were treated—29 with acetaminophen and 32 with ibuprofen. These 61 patients were categorized into two groups based on the consistency of the drug regimen between the first and second courses; the first group received the same drug in both courses, while the second group received a different drug in the second course (e.g., acetaminophen in the first course and ibuprofen in the second, or vice versa).

The ductal closure rate in the first group (same treatment in both courses) was 62.2%, while the closure rate in the second group (switch of treatment between courses) was 70.7%. No statistically significant differences were recorded between these groups (Table 4 and Table 5).

## 4. Discussion

Through our comprehensive, single-center retrospective analysis, conducted over 10 years, we delineated the comparative effectiveness of two distinct pharmacological interventions for the closure of hemodynamically significant patent ductus arteriosus (hsPDA). Our findings revealed no statistically significant differences in the efficacy of acetaminophen versus ibuprofen, although a marginal numerical advantage was observed with acetaminophen. Furthermore, we demonstrated that administering a second course of pharmacological therapy could effectively induce hsPDA closure, thereby potentially obviating the necessity for invasive surgical procedures.

This study aimed to determine the effectiveness of a second pharmacological approach in inducing hsPDA closure when the initial treatment failed. The persistence of hsPDA refractory to first-line pharmacological intervention often presents clinicians with the clinical conundrum of either adopting a strategy of watchful waiting for spontaneous ductal closure or proceeding with invasive surgical intervention to mitigate hsPDA-associated risks [2,5,6,7,20], while acknowledging the potential adverse effects of surgical procedures [1].

We stratified patients who received a second course of pharmacological treatment into two groups: the first group comprised patients treated with the same drug in both courses, and the second group consisted of patients treated with different drugs between the first and second courses. Our findings indicated a numerically higher success rate for hsPDA closure with pharmacological approach variation, although this difference did not reach statistical significance. Considering all patients who achieved hsPDA closure after either the first or second course of therapy, our cohort’s overall pharmacological treatment success rate was 92%. Among the 21 patients who did not achieve closure after two courses, 16 underwent surgical intervention. While surgical intervention is definitive, it carries inherent risks, particularly in low-birth-weight infants [1]. A watchful waiting approach until patients reach a suitable weight for surgery may prolong exposure to hsPDA-related complications, potentially leading to fatal outcomes [3,10,23,24].

Numerous studies [8,9,13,14,15,16,21,25,26,27,28] have compared the efficacy of ibuprofen and acetaminophen in terms of hsPDA closure. Our data are consistent with the existing literature, demonstrating comparable efficacy for both pharmacological approaches in achieving hsPDA closure after the first course of therapy. Several studies aligning with our findings [9,28], as well as data on the closure effectiveness of a second course of medical therapy, should be reported.

However, no prior study has directly compared the outcomes of a second course using a drug different from the initial regimen. The rationale for changing therapy after initial failure stems from the distinct pharmacological mechanisms of action of ibuprofen and acetaminophen in modulating ductus arteriosus patency, as described by Jasani [29]. Although ibuprofen and acetaminophen share functional similarities, they differ in their mechanism of action. Ibuprofen acts on the cyclooxygenase (COX) site of the enzyme prostaglandin H2 synthetase to reduce prostaglandin production, while acetaminophen is thought to act through the peroxidase (POX) [29].

A well-designed study by Moghtaderi [28] compares the combined use of ibuprofen and paracetamol in closing the ductus arteriosus, with higher success rates than in monotherapy, at least in the second course.

The two cohorts differed slightly in median gestational age and birth weight, with lower values observed in the acetaminophen group. We speculate that this finding may reflect improved management of extremely preterm infants, including those born at less than 24 weeks gestation. Despite this disparity, no statistically significant differences were observed between the treatment groups regarding the incidence of BPD, spontaneous intestinal perforation, or ROP requiring treatment. We identified a statistically significant difference only in the incidence of NEC, which was markedly lower in the acetaminophen group.

In our interpretation, this difference is more likely attributable to implementing our internal feeding protocol for VLBW infants we adopted in 2019, which emphasizes a more cautious approach to enteral feed advancement, rather than a direct effect of ibuprofen treatment. Furthermore, no statistically significant differences were noted in the incidence of severe brain injury (IVH, CBH, and PVL).

Although patients with lower gestational age and birth weight were more likely to experience failure after the first course of pharmacological therapy, these factors did not significantly influence the outcomes of second-line treatment. This subgroup is known to have an increased risk of developing complications such as BPD and ROP, as also observed in our cohort. However, these outcomes may be more directly influenced by the hemodynamic instability and oxidative stress associated with persistent PDA rather than by the pharmacological response itself [3,4]. These findings suggest that, even in clinically fragile neonates, a second course of medical therapy remains a viable and potentially effective option for achieving ductal closure.

In our data, patients who fail the initial pharmacological therapy for PDA closure present with a lower gestational age and birth weight.

A unique strength of our study is the routine performance of brain MRI at corrected term age in all VLBW infants. This enabled us to compare the two groups for macroscopic lesions detectable by ultrasound and subtle hemorrhagic lesions (using susceptibility-weighted imaging) and punctate white matter lesions associated with prematurity. Identifying treatment-related differences in the incidence of these lesions is clinically relevant for guiding therapeutic decisions, as they are known risk factors for neurodevelopmental impairment. Despite a lower percentage of minor IVH in the ibuprofen group, no statistically significant differences were observed.

Conflicting reports exist regarding the long-term outcomes of perinatal and early neonatal acetaminophen exposure. At the same time, observational studies have suggested associations between prenatal acetaminophen exposure and long-term developmental and behavioral disorders. A sizeable neonatal cohort study found no association between intravenous acetaminophen exposure and morbidities at 5 years of age [14].

Our data indicate comparable efficacy between acetaminophen and ibuprofen for hsPDA closure in preterm neonates born at less than 32 weeks gestation, in both first-line and second-line therapy. We also demonstrated that switching drugs between the first and second courses did not significantly improve hsPDA closure rates. Although our study demonstrates the non-inferiority of acetaminophen to ibuprofen in hsPDA closure, clinical scenarios may exist where one agent is preferred. Ibuprofen, while effective, carries a risk of renal side effects and gastrointestinal bleeding [8,13,14,15,16,17], making acetaminophen a potentially more favorable first-line agent in patients with pre-existing renal dysfunction or increased risk of hemorrhage. Conversely, in cases where rapid ductal closure is imperative, some studies report the faster onset of action for ibuprofen (within the third dose administration/third day of treatment) [12]. This might make it the preferred choice.

The primary limitation of our study is its retrospective design. Although the database from which patients were selected was meticulously compiled, retrospective data entry may introduce inaccuracies. Furthermore, our retrospective study design limits our ability to assess the incidence and severity of drug-related side effects comprehensively. We did not systematically collect data on renal function or detailed information on gastrointestinal events, which would be necessary for a thorough safety comparison. Future prospective studies should include rigorous monitoring for these potential adverse effects.

## 5. Conclusions

In conclusion, this study confirms that acetaminophen is non-inferior to ibuprofen in achieving hsPDA closure in preterm neonates born at less than 32 weeks gestation. Our analysis of second-line therapy outcomes suggests that acetaminophen and ibuprofen have comparable efficacy overall. We also demonstrated that changing the drug between the two courses does not significantly enhance hsPDA closure rates. However, the trend towards improved acetaminophen response in moderate hsPDA after first-line failure warrants further investigation. This finding implies that in cases of persistent moderate hsPDA, acetaminophen might be a reasonable second-line option, potentially delaying or avoiding surgical intervention.

Future studies should prospectively evaluate this observation and explore other factors influencing second-line treatment success, such as the initial treatment duration and specific patient comorbidities.

## Figures and Tables

**Table 1:** Clinical characteristics of patients treated for hsPDA (first course of pharmacological therapy).

	Acetaminophen	Ibuprofen	*p*-Value
patients	171	115	
median gestational age (IQR)	27 + 5 (3.7)	28 + 1 (2.6)	0.01
median birth weight (IQR)	940 g (470)	1025 g (425)	0.03
BPD (%)	66/171 (39.0%)	55/115 (47.8%)	0.17
NEC (%)	6/171 (3.5%)	13/115 (11.3%)	0.01
SIP (%)	12/171 (7.3%)	4/115 (3.4%)	0.14
treated ROP (%)	38/171 (22.3%)	33/115 (28.2%)	0.23
any grade IVH (%)	40/171 (23.7%)	18/115 (15.6%)	0.12
severe IVH (%)	14/171 (8.1%)	7/115 (6.2%)	0.42
massive CBH (%)	4/171 (2.4%)	2/115 (1.7%)	0.42
punctate white matter lesions (%)	13/171 (7.6%)	10/115 (8.7%)	0.77
PVL (%)	2/171 (1.3%)	2/115 (1.7%)	0.82

IQR, inter-quartile range; BPD, bronchopulmonary displasia; NEC, necrotizing enterocolitis; SIP, single intestinal perforation; ROP, retinopathy of prematurity; IVH, intraventricular hemorrhage; CBH, cerebellar hemorrhage; PVL, periventricular leukomalacia.

**Table 2:** Clinical characteristics of patients treated for hsPDA (second course of pharmacological therapy).

	Acetaminophen	Ibuprofen	*p*-Value
patients	29	32	
median gestational age (IQR)	26 + 5 (2)	27 + 2 (3.5)	0.88
median birth weight (IQR)	855 g (240)	895 g (315)	0.26
BPD (%)	14/29 (48.3%)	18/32 (51.4%)	0.55
NEC (%)	1/29 (3.5%)	4/32 (11.3%)	0.07
SIP (%)	2/29 (6.8%)	0/32 (0%)	0.10
treated ROP (%)	7/29 (24.1%)	11/32 (31.3%)	0.25
any grade IVH (%)	5/29 (17.2%)	11/32 (34.3%)	0.11
severe IVH (%)	2/29 (6.8%)	1/32 (3.1%)	0.60
massive CBH (%)	1/29 (3.4%)	0/32 (0%)	0.33
punctate white matter lesions (%)	2/29 (6.8%)	3/32 (9.3%)	0.77
PVL (%)	0/29 (0%)	1/32 (3.1%)	0.30

IQR, inter-quartile range; BPD, bronchopulmonary displasia; NEC, necrotizing enterocolitis; SIP, single intestinal perforation; ROP, retinopathy of prematurity; IVH, intraventricular hemorrhage; CBH, cerebellar hemorrhage; PVL, periventricular leukomalacia.

**Table 3:** A comparison of patients who achieved hsPDA closure after the first drug course with those who experienced treatment failure.

	Failure	Success	*p*-Value
patients	61	225	
median gestational age (IQR)	26 + 5 (2)	28 + 2 (3)	<0.01
median birth weight (IQR)	880 g (260)	895 g (485)	<0.01
BPD (%)	14/29 (65.5%)	24/225 (36.0%)	0.01
NEC (%)	1/29 (10.6%)	4/32 (6.1%)	0.25
SIP (%)	2/29 (3.5%)	0/32 (7.0%)	0.36
treated ROP (%)	5/29 (39.6%)	18/32 (21.0%)	<0.01
any grade IVH (%)	5/29 (25.0%)	11/32 (20.1%)	0.33
severe IVH (%)	2/29 (8.6%)	1/32 (6.5%)	0.57
massive CBH (%)	1/29 (1.7%)	0/32 (1.5%%)	0.88
punctate white matter lesions (%)	2/29 (6.8%)	3/32 (6.1%)	0.77
PVL (%)	0/29 (1.7%)	1/32 (1.5%)	0.90

IQR, inter-quartile range; BPD, bronchopulmonary displasia; NEC, necrotizing enterocolitis; SIP, single intestinal perforation; ROP, retinopathy of prematurity; IVH, intraventricular hemorrhage; CBH, cerebellar hemorrhage; PVL, periventricular leukomalacia.

**Table 4:** Efficacy of first and second course of pharmacological therapy in hsPDA closure.

Closure Rate (%)	Acetaminophen	Ibuprofen	*p*-Value
first course	138/171 (80.7%)	87/115 (75.7%)	0.55
second course	20/29 (69.2%)	20/32 (62.5%)	0.56

**Table 5:** Closure rate after second course of pharmacological therapy.

	Same Drug	Different Drug	*p*-Value
closure rate after second course of pharmacological therapy (%)	17/24 (62.2%)	23/37 (70.7%)	0.45

## Data Availability

The data presented in this study are available on request from the corresponding author due to privacy restricions.
